# A direct, sensitive and high-throughput genus and species-specific molecular assay for large-scale malaria screening

**DOI:** 10.1186/s40249-022-00948-2

**Published:** 2022-03-07

**Authors:** Yaling Zhao, Ye Zhao, Yu Sun, Lihua Fan, Duoquan Wang, Heng Wang, Xiaodong Sun, Zhi Zheng

**Affiliations:** 1 Institute of Basic Medical Sciences, Chinese Academy of Medical Sciences, School of Basic Medicine, Peking Union Medical College, Beijing, China; 2 Yunnan Institute of Parasitic Diseases, Yunnan Center for Malaria Research, Institute of Vector and Pathogen Biology, Dali University, Puer, Yunnan China; 3 National Institute of Parasitic Diseases, Chinese Center for Disease Control and Prevention, WHO Collaborating Center for Malaria, Schistosomiasis, and Filariasis, Key Laboratory of Parasite and Vector Biology, Ministry of Health, Shanghai, China; 4 Blood Transfusion Department, Peking Union Medical College Hospital, Chinese Academy of Medical Sciences, Beijing, China

**Keywords:** Infectious disease, Malaria, Molecular screening, CLIP-PCR, Genus, Species, High-throughput

## Abstract

**Background:**

Infectious disease diagnostics often requires sensitive molecular assays that identify at both genus and species levels. For large scale screening, such as malaria screening for elimination, diagnostic assay can be a challenge, as both the throughput and cost of the assay must be considered. The requirement of nucleic acid extraction hampers the throughput of most molecular assays. Co-amplification of multiple species or multiplex identification either can result in missed diagnosis or are too costly for large-scale screening. A genus- and species-specific diagnostic assay with simplified procedure, high sensitivity and throughput is still needed. This study aimed to develop a sensitive and high-throughput approach for large-scale infectious disease screening.

**Methods:**

We developed multi-section Capture and Ligation Probe PCR (mCLIP-PCR) for the direct detection of RNA without extraction and reverse transcription. Multiple tailed sandwich hybridization probes were used to bind at genus- and species-specific sections of the target RNA to cooperatively capture the target onto a 96-well plate. After enzymatic ligation of the bound probes, a single-stranded DNA formed at each section with distinct tail sequence at the ends. They were separately PCR-amplified with primers corresponding to tail sequences for genus or species identification. We applied the method to the active screening of *Plasmodium* infections of 4,580 asymptomatic dried blood spot samples collected in malaria endemic areas and compared the results with standard qPCR using linear regression.

**Results:**

With multi-section cooperative capture but separate amplification strategy, we accurately identified genus *Plasmodium* and species *P. falciparum* and *P. vivax* without RNA extraction, with favorable sensitivities among the published reports. In the active screening, our method identified all 53 positive infections including two mixed infections, and two *P. vivax* infections that were missed by standard qPCR.

**Conclusions:**

mCLIP-PCR provides a sensitive and high-throughput approach to large-scale infectious disease screening with low cost and labor, making it a valuable tool for malaria elimination in endemic region.

**Graphical Abstract:**

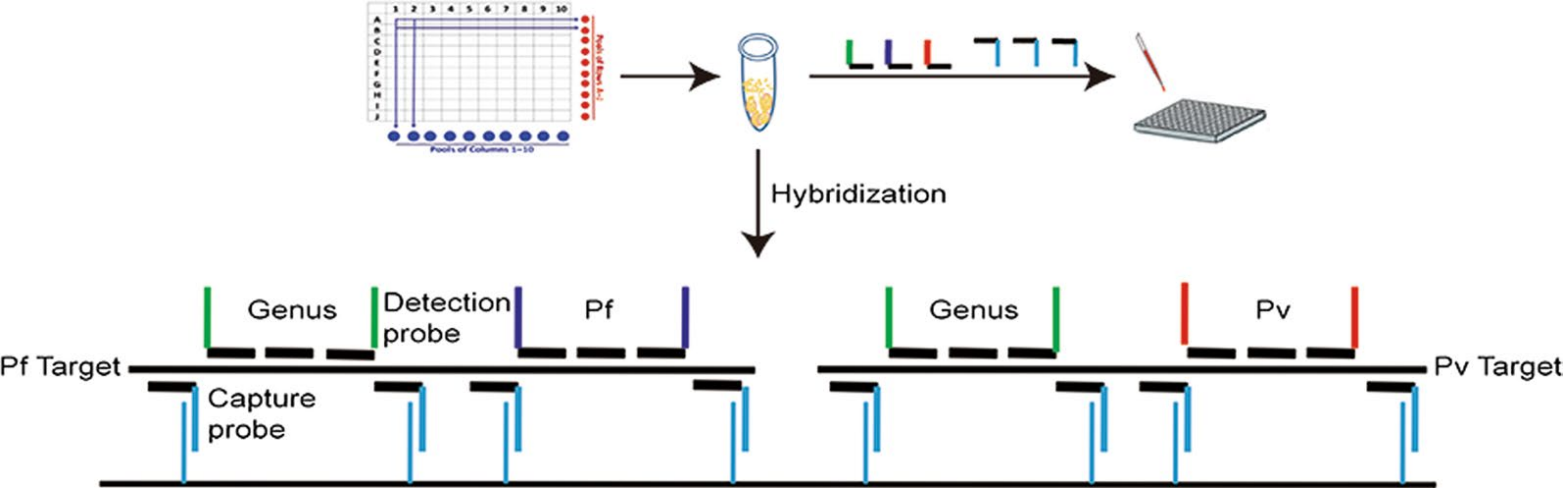

**Supplementary Information:**

The online version contains supplementary material available at 10.1186/s40249-022-00948-2.

## Background

In clinical microbiology, diagnostics at both the genus and species levels are often required. As an example, *Plasmodium falciparum* and *P. vivax* are the two most prevalent *Plasmodium* species responsible for the majority of malaria cases in the world [1]. It is crucial to differentiate these *Plasmodium* species as they require different therapies. Mixed infections in the same individual are not uncommon. Surveys usually report that about 2% of infections are mixed, or 5–30% with more sensitive detection methods [2]. These mixed infections are often missed in clinical practice leading to incorrect treatments that result in severe consequences [2, 3]. Missed *P. falciparum* infection results in the risk of developing subsequently severe, possibly fatal disease; while missed *P. vivax* infection could result in repeated debilitating disease [1, 4]. Therefore, accurate diagnosis at both genus and species level are essential.

Both microscopy and rapid diagnostic tests (RDTs) have insufficient sensitivities for low parasitemia (< 100 parasites/μl) and mixed parasite infections [5, 6], with the limit of detection of about 50‒499 parasites/μl for light microscopy, and about 200 parasites/μl for RDT [7]. As a result, poor agreement (69%; kappa = 0.56) among microscopists was reported for the detection of parasites at a low density and mixed infections [8]. Molecular methods are commonly used for species identification, particularly for mixed infections [9–17]. These methods are relatively straightforward when only a limited number of specimens are involved in each run. However, for screening applications involving a large number of samples, such as active case detection for elimination, diagnostic assay can be a challenge as both the throughput and cost of the assay have to be considered [7]. Conventional methods of *Plasmodium* screening usually contain two testing steps: a genus amplification for the first round of a screening run, and with genus-positives, a subsequent assay for species identification [9, 10, 18]. For example, one study [10] used the widely-used genus-specific primers from a classical nested PCR detection [9] to amplify all plasmodia parasites in the first PCR, followed by separate amplifications of individual species from the first round positive PCR products using nested species-specific primers. For screening operations this 2-tiered approach has the obvious advantage of being most economical. However, for mixed infections, it often misses the identification of minor species, since in the first PCR with the common primers, the predominant species may amplify first and subsequently monopolize the PCR [1, 4, 12]. To overcome this, direct species-amplification is often used where at least one primer is species-specific. Multiplex PCR approaches have been developed [12, 13, 16, 17, 19, 20] that allow the detection of several species in one reaction, leading to the simplified work flow. But the multiplex assays always rely on specific fluorescence multi-color probes or agarose gel electrophoresis that may be either too costly or labor intensive for large-scale screenings, especially in resource-limiting situations [9, 21].

For infectious disease screening, nucleic acid extraction remains the most significant impediment to increased throughput and reduced cost and labor. A “direct PCR” approach with no need for DNA isolation has been developed based on genetically modified DNA polymerase that are resistant to polymerase inhibitors in blood [18, 22–25], facilitating high-throughput analysis of samples collected in the field [24, 25]. However, the proprietary and costly reagents greatly limit its large-scale application; moreover, the modified DNA polymerases may be insufficiently active in the presence of high volumes of blood, the limit of which is sample dependent [18, 25, 26]. This often resulted in reduced sensitivity compared with using purified DNA [24]. The presence of blood in the PCR reaction may also impact downstream real-time fluorescence detection and compromise sensitivity [24, 27, 28].

Inspired by protein immunoassays such as enzyme linked immunosorbent assay (ELISA), whereby no target purification is required and a sandwich assay is used to capture specific target onto a solid surface for subsequent detection, we previously developed a nucleic acid detection assay called Capture and Ligation Probe-PCR (CLIP- PCR), which bypasses nucleic acid purification and reverse transcription while achieving RT-PCR sensitivity [29]. In CLIP-PCR, oligo probes analogous to capture and detection antibodies bind and capture the target nucleic acid onto solid support. Subsequent PCR serves to exponentially amplify the detection signal, analogous to the enzymatic cascade reaction that amplify the detection signal in immune assays. As a powerful high throughput RNA quantification technology, CLIP-PCR is ideal for first round genus screening, subsequent species identification, however, relies on a separate quantitative PCR (qPCR) starting from DNA extraction from the original blood or dry blood spot (DBS) samples [29]. Also, an overnight incubation step in CLIP-PCR prolongs the turnaround time. Therefore, a rapid, low-cost, high-throughput and sensitive assay for both genus and species screening is still needed.

Unlike proteins, which are mostly globular and compact, nucleic acid targets assume more extended conformations at elevated temperatures and are therefore amenable for sandwich capture at multiple sections of the same target molecule. By simultaneous sandwich hybridization capture at multiple sections of a target molecule, instead of one section only, more efficient capture can potentially be realized. Here we describe a multi-section CLIP-PCR strategy (mCLIP-PCR) to capture both genus and species-specific sequences of target pathogens simultaneously in the same well for subsequent detection. The assay screens for genus *Plasmodium* infections in the first round; and with positives, continues with species qPCR runs without additional sample processing, and without amplification dropout. The strategy significantly improves the assay efficiency, resulting in a total assay time of about 4 h instead of overnight. Absent of RNA purification and reverse transcription, our assay is fast and convenient as a highly sensitive and high-throughput approach for genus *Plasmodium*18S rRNA and species *P. falciparum* and *P. vivax* 18S rRNA screening with low cost and labor.

## Methods

### Blood samples

The samples were collected near Lazan City, Myanmar along the China-Myanmar border from March 28, 2017 to May 23, 2017. Local malaria transmission season begins from May to October. DBS (from about 50 μl finger prick blood) from asymptomatic villagers (body temperature less than 37.8 °C) were prepared on Whatman 3MM filter papers according to standard protocol [30], and within 7 days stored with desiccant at − 20 °C. Samples were sent in batches to a screen lab in the Nabang Township Hospital in the adjacent Chinese border town of Nabang in Yingjiang County, Yunnan Province. The DBS samples were dried in the lab at room temperature overnight before test. Blood samples taken from healthy individuals without any travel history to malaria endemic areas were used as negative controls.

### DBS sampling and pooling

A 3 mm-diameter disc was punched from the DBS using a handheld metal hole-puncher. To prevent carryover contamination, after each punch the puncher head was dipped with 70% alcohol, flamed over a Bensen burner, and let cooled down before next use. A matrix-pooling protocol [29] were used for sample pooling. Briefly, samples were randomly arranged in sets of M by M matrix, each sample was punched and pooled with samples in the same row, and separately with samples in the same column. Such M-pooled samples were lysed and tested as one sample by mCLIP-PCR first-round genus assay. In this way, each sample was tested once in a row pool and once in a column pool, and a positive sample would give rise to corresponding row pool and column pool positives. Conversely, however, samples at the intersections of positive row pools and positive column pools may or may not be positive (in cases when more than one intersection appear in a positive row or column) and will be tested further, while all others were declared negative. Pooling was done by groups of two individuals, with one performing the punch while the other stood by with careful monitoring, sample tracking and note taking to ensure proper handling.

### DNA extraction, standard quantitative PCR (qPCR) and sequencing

DNA was extracted from 200 μl of thawed EDTA-blood or four 3 mm DBSs with the TIANamp Blood or Blood Spots DNA Kits (TIANGEN, Beijing, CHINA) according to the manufacturer’s instructions. The extracted DNA was dissolved in 35 μl ddH_2_O and quantified with NanoDrop (Thermo Fisher, Waltham, USA) and stored at – 20 °C. The standard quantitative TaqMan PCR (qPCR) was performed on 40 ng DNA with a LightCycler^®^ 96 (Roche), the genus qPCR primers were derived from the reference [31] and included a forward primer (5′-GTTAAGGGAGTG AAGACGA TCAGA-3′), a reverse primer (5′-AACCCAAAGACTTTGATTTC TCATAA-3′) and probe (5′-FAM-ACCGTCGTAA TCTTAACCAT AAACTATGCC GAC TAG-TAMRA-3′). *P. falciparum* and *P. vivax* assays were performed in two separate reactions as reported [32]. At least three positive and negative controls were included to each experiment. Samples with a cycle threshold (CT) ≤ 40 were considered positive.

For species identification by sequencing, DNA was amplified with the genus primers [31], and subjected to Sanger sequencing on an ABI3100 capillary sequencer (Applied Biosystems, Waltham, USA).

### Quantification of human malaria parasites using droplet digital PCR (ddPCR)

To determine the analytical sensitivity of the assays, we used serial dilutions of standard samples whose parasite densities were determined by the method of ddPCR [33]. Briefly, DNA was extracted from either a *P. falciparum* blood culture or a *P. vivax* blood sample, each sample was partitioned into approximately 10,000 droplets and the 18S rRNA gene was amplified and quantified using probes and primers derived from genus screening [31] on a Bio Rad QX200 droplet PCR system. The number of droplets with amplification product was then measured, allowing for an estimate of template density without the need for a standard curve [33].

### Multi-section CLIP-PCR (mCLIP-PCR) for genus and species determination

Sample (three 3 mm-DBS or 72 μl whole blood) was added to a total volume of 360 μl (3 replicates) with 120 μl lysis mixture (Diacurate, Beijing, China), 16.8 μl proteinase K (20 mg/ml), 3.6 μl genus probe mix, 3.6 μl species capture probe mix, 7.2 μl species detection probe mix and water. The whole mix was vigorous shaken (800 rpm) at 56 °C for 30 min, and 100 μl/well was transferred to a 96-well capture plate. After mild shaking (300 rpm) at 55 °C for 30 min, each well was washed for three times with 150 μl wash buffer [0.1 × Saline Sodium Citrate (SSC), 0.1% SDS], followed by addition of 50 μl ligation mix (New England Biolab, Ipswich, USA) for 30 min ligation at 37 °C. The ligated product was heat-released from plate at 90 °C for 1 min, and 5 μl was transferred to a new PCR plate for the genus qPCR run in a total of 25 μl/well containing 1 × SYBR^®^ Premix Ex (Takara) and 100 nmol/L genus primers. For genus positives, another 5 μl of ligation product was amplified in separate *P. falciparum* or *P. vivax* qPCR runs in 25 μl/well containing 1 × SYBR^®^ Premix Ex (Takara) and 200 nmol/L corresponding species primers. The assay was performed on a LightCycler^®^ 96 Real-Time PCR System (Roche, Basel, Switzerland) with the following thermal profile: 95 °C for 30 s, 45 cycles of 95 °C for 5 s, 60 °C for 20 s, 72 °C for 20 s. The specific product was defined by melting curve analysis. The sample was considered positive if the melting curve was the same as that of the positive control.

For analyzing the sensitivity of our assays, we used a sample of *P. falciparum* 3D7 ring-stage synchronized culture and a whole blood sample infected by *P. vivax*. The parasite density of each sample was first determined by ddPCR. We then took threefold serial dilutions, and each parasite concentration was assayed with three replicates and repeated on three different days to determine the detection limit of the genus and species assays.

**Fig. 1:**
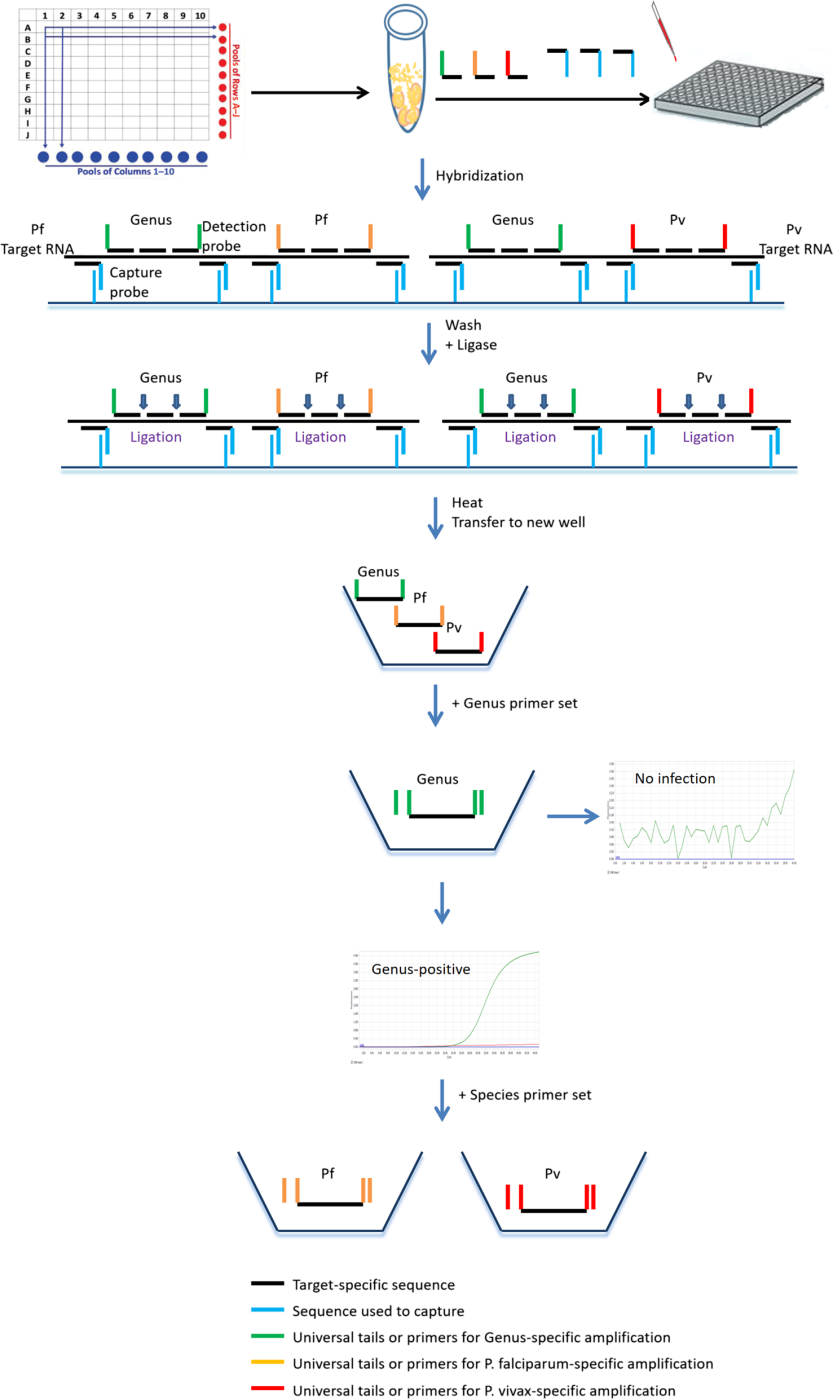
Workflow of multi-section CLIP-PCR assay (mCLIP-PCR) for *Plasmodium* genus and species screening. Dried blood samples are first pooled and lysed. Depicted here is a 10 × 10 matrix-pooling, where samples in the same row are pooled and tested together as one, and so are the samples in the same column. In this way all 100 samples are analyzed twice just by testing Pool A to J and Pool 1–10 (in a total of 20 tests). The released target RNAs from both *P. falciparum* (Pf) and *P. vivax* (Pv) are captured to the same well in a 96-well plate via hybridization with oligo probes on multiple sections of each target RNA. Sections include either conserved (“Genus”) or species-specific (“Pf” or “Pv”) sequences of each target RNA. Within each section, probes bind contiguously to the target. Capture Probes (which includes a target-specific sequence and a common tail sequence) at the two ends of a section anchors the target to solid surface by hybridizing the tail sequence with oligos conjugated on the solid surface. After washing, the Detection Probes between the two Capture Probes are ligated to form a single-stranded DNA in each section with both ends having defined sequences as corresponding PCR primer sites. The ligation products are heat-released from the well bottom and 5 μl of which transferred to a new plate for genus PCR amplification using genus-specific primer set (green) and SYBR green chemistry. For the genus-positive sample, additional 5 μl of released ligation product is separately amplified by *P. falciparum*- or *P. vivax*-specific primer set (colored orange and red respectively)

To test for potential cross-reactivity of capture probes or PCR primers, we prepared three samples each of *P. falciparum* (8 parasites/μl), *P. vivax* (8 parasites/μl), an equal mix of each (as mock mixed infection), and negative control blood. Each sample was lysed to release the RNA targets, which were captured by all hybridization probes (capture probes and detection probes) for genus and species (Fig. 1). After ligation of the bound detection probes, the ligated product was separately amplified by each of the three sets of primers corresponding to the tail sequence of detection probes (genus, species *P. falciparum* and *P. vivax*) (Fig. 1).

We prepared mock mixed infections by artificially mixing infected blood containing *P. falciparum* and *P. vivax* at different parasite ratios of 1:1, 1:3, 1:5, 1:7 respectively, each original *P. falciparum* and *P. vivax* infected blood contain about 30 parasites per microlitre. These mock mixed infections as well as healthy control blood were analyzed by mCLIP-PCR species assay and by standard qPCR species assay after DNA extraction.

### Statistical analysis

The Cq values from mCLIP-PCR and standard qPCR were presented as mean ± standard deviation (*SD*). Calculations of sensitivity, specificity and agreement between mCLIP-PCR and standard qPCR were done using SPSS 22.0 (IBM, New York, USA); kappa coefficient values express the agreement beyond chance and were calculated with a 95% confidence interval.

## Results

### mCLIP-PCR for both *Plasmodium* genus and species detection

Figure 1 illustrates the principle of the mCLIP-PCR workflow. For genus and species *Plasmodium* detection, we designed different sets of oligo nucleotide probes to 18S ribosomal RNA (18S rRNA, GenBank accession numbers: *P. falciparum*: M19172.1; *P. vivax*: U03079.1; *P. malariae*: AF488000.1; *P. ovale*: L48987.1; *P. knowlesi*: L07560.1), targeting conserved regions for the *Plasmodium* genus and species-specific region for *P. falciparum* and for *P. vivax* (Fig. 1 and Additional file 1: Table S1). In addition to target-specific sequence, most probes contain designed “tail” sequences that are independent of the target sequence but can interact with either the solid support or the detection system (Fig. 1). The assay involves first capturing the 18 s rRNA of all five *Plasmodium* species in the sample to the bottom of the plate well through multi-section sandwich hybridization, followed by probe ligation and genus detection in the first round of qPCR, and species identification in the 2nd round of qPCR. The first-round workflow, from the start of sample lysis to the genus detection result, took about 3 h with minimal hands-on time. Additional species determination took about another 1 h, for a total of 4 h. Prior to the qPCR, the ELISA-like workflow in 96-well plate format involved only rounds of reagent addition, incubation, and wash, without RNA purification or reverse-transcription.

### Analytical performance of the genus assay and the species assays

**Fig. 2:**
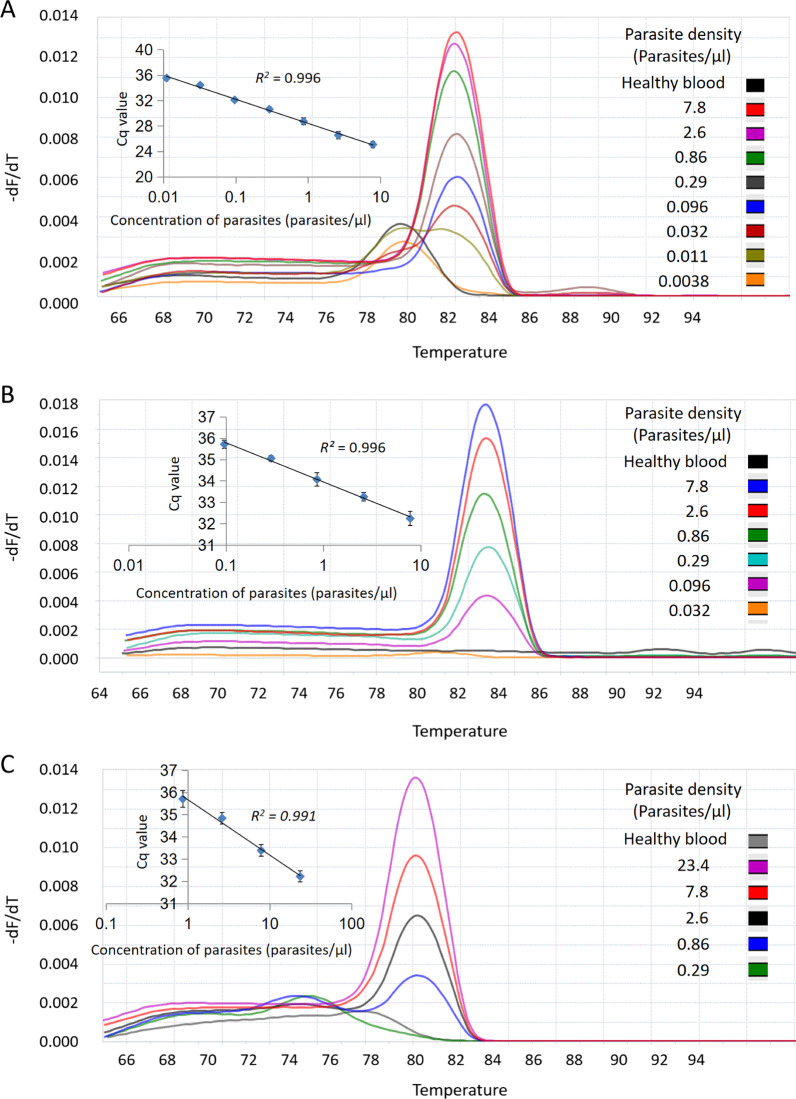
Analytical performance of mCLIP-PCR for both genus and species detection. *P. falciparum* or *P. vivax* samples were threefold serially diluted and tested by multi-section CLIP-PCR in triplicates in three independent experiments (9 duplicates total). Representative PCR product melting curves, and the Cq vs parasite concentration plots, were shown for genus (**A**), *P. falciparum* (**B**), and *P. vivax* (**C**)

**Fig. 3:**
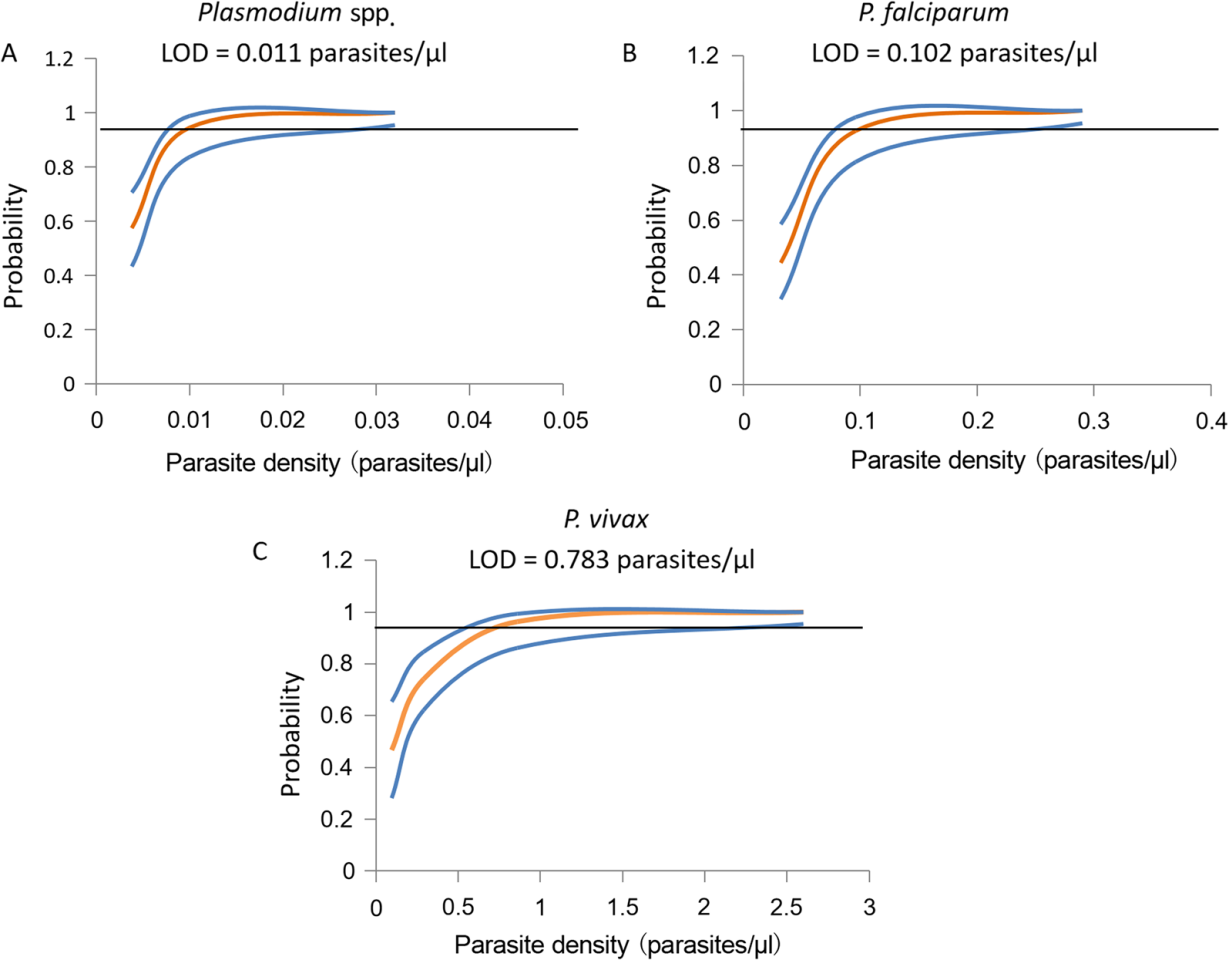
Analytical sensitivity of mCLIP-PCR determined by probit regression. LOD with 95% confidence intervals was analyzed using probit analysis with MedCalc 16.4 (MedCalc Software, Ostend, Belgium). For each curve a total of 54 replicates were run at each concentration approaching the LOD. The red line depicts the regression curve and the blue line represents the 95% confidence intervals. *LOD* Limit of detection.

The results of our assays showed the positive melting peak can be clearly distinguished from that of the primer dimer (Fig. 2). The mean of the quantification cycle (Cq) values vs concentration generated linear standard curves with R^2^ > 0.98 (Fig. 2). To more accurately determine the limit of detection (LOD), we used probit analysis [34]. At concentrations approaching the LODs estimated from Fig. 2, we run additional six samples, each in three replicates in three independent experiments, totaling 54 replicates (Additional file 2: Table S2). The LOD with 95% confidence intervals by probit analysis was 0.011 parasites/μl (0.0086–0.029) for *Plasmodium* spp., 0.102 parasites/μl (0.078–0.278) for *P. falciparum*, and 0.783 parasites/μl (0.579–2.234) for *P. vivax* (Fig. 3).

In the test for potential cross-reactivity of capture probes or PCR primers, we found positive specific signals only when the corresponding species was present in the reaction, giving an analytical specificity of 100%. The results of standard qPCR using purified DNA showed the same differentiation.

### Singleplex vs multiplex determination of species in mock mixed infections

**Fig. 4:**
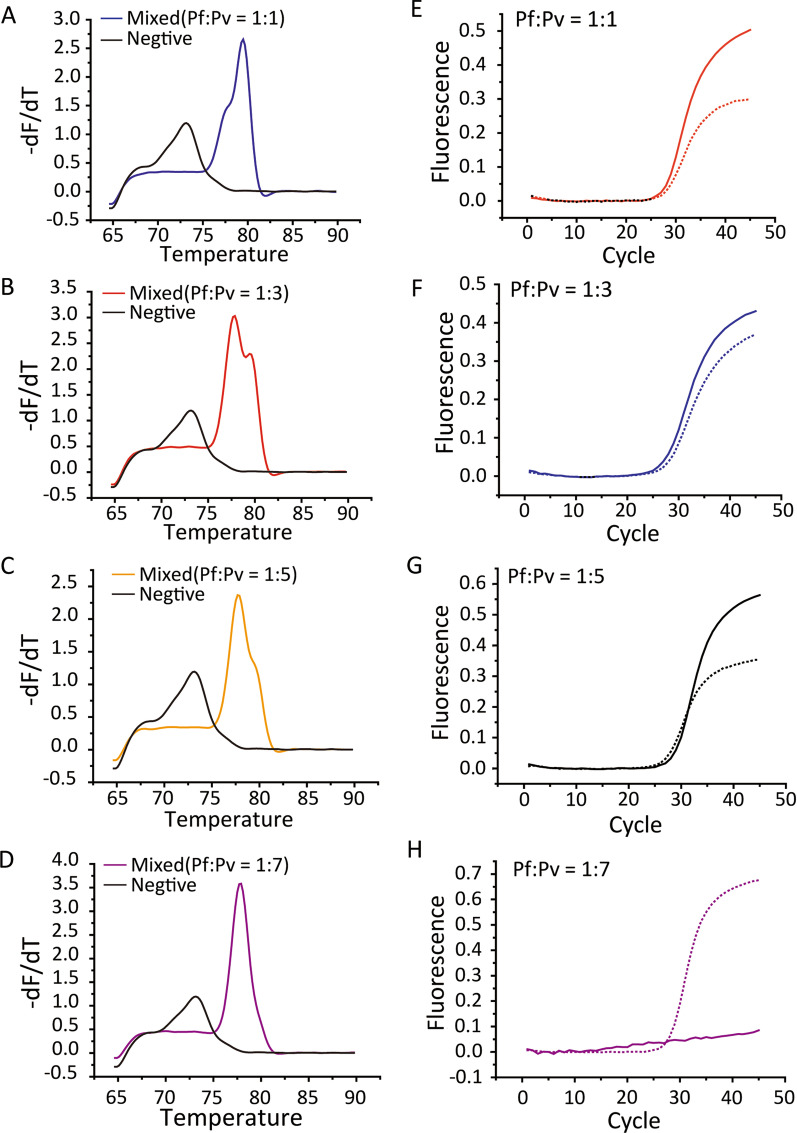
Multiplex assays of mCLIP-PCR and TaqMan on mock mixed infections. Mixed blood with low concentrations ratios of *P. falciparum* (Pf) to *P. vivax* (Pv) were detectable by multiplex mCLIP-PCR (**A**–**C**) and TaqMan multiplex assays (**E**–**G**), while at higher ratio only predominant species can be detected (**D** and **H**). Solid lines in TaqMan refer to the *P. falciparum*, while dotted lines refer to *P. vivax*. The figure is representative of 3 independent experiments

Assays of all the mock mixed infections showed correct species identification in singleplex assays of mCLIP-PCR and TaqMan qPCR (Additional file 3: Table S3). When we attempted multiplex species amplification of the ligated products with two pairs of primers in one well, two parasites can be simultaneously detected only when the ratio is relatively low (Fig. 4A‒C), and at high concentration ratio only the predominant species can be detected (Fig. 4D). Multiplex standard qPCR on the same series produced similar results (Fig. 4E–H). While it may be possible by PCR optimization to achieve balanced amplification at higher ratios, the possibility that dropout may occur after certain ratio made us decide that the species detection in mCLIP-PCR assay be run individually rather than multiplex.

### Genus and differential *Plasmodium* species screening with field samples

**Fig. 5:**
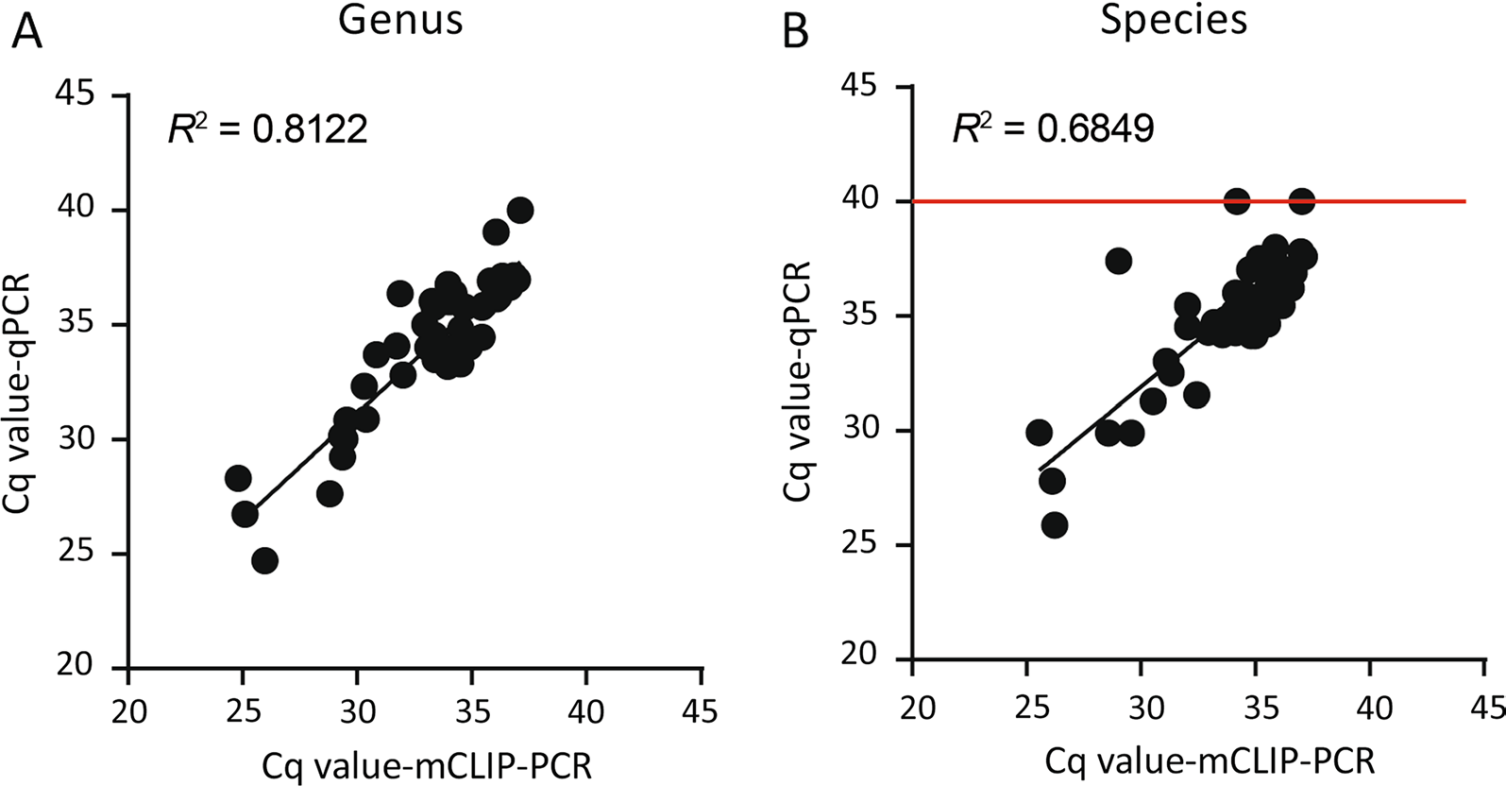
Correlation between mCLIP-PCR and singleplex TaqMan qPCR. The Cq values of the 53 genus-positive samples as determined by genus (**A**) and species-specific (**B**) assays of mCLIP-PCR and qPCR were plotted. Redline in **B** indicates sample negativity in qPCR

We screened 4,580 DBSs collected from asymptomatic donors in malaria-endemic region with a matrix-pooling strategy (Fig. 1). The mean number of DBS per pool (M) was 16 (a 16 × 16 matrix can screen 256 samples in a total of 32 pooling tests). About 576 pools were tested in the first round of genus screening, resulting in a total of 100 individual samples at the intersections of positive row and column pools. These 100 candidate positive samples (which may include positives or negatives) were further tested for *Plasmodium* genus, *P. falciparum* and *P. vivax* infections using mCLIP-PCR and singleplex qPCR. Our mCLIP-PCR identified 14 *P. falciparum* infections, 35 *P. vivax* infections and 2 mixed infections (both *P. falciparum* and *P. vivax* positive) (Table 1). There were two genus-positive samples at low parasitemia (mean Cq = 36.67 in genus assay) that were not identifiable at species level in our assay, and both turned out to be *P. vivax* samples by sequencing. All 100 samples were also examined by standard singleplex qPCR after DNA extraction, using genus and species-specific primers and TaqMan probes. The standard TaqMan qPCR, like mCLIP-PCR, also showed higher sensitivity for genus than for species detection, giving four samples that were genus-positive only (Table 1). Compared with mCLIP-PCR, the TaqMan assay failed to identify 2 more *P. vivax* samples at species level (Table 1) which were confirmed by sequencing. Correlations between Cq values of mCLIP-PCR and Taqman qPCR were high (Fig. 5).

**Table 1:** Number of samples identified by *Plasmodium* genus and species assays*

Genus/species	mCLIP-PCR	qPCR (singleplex)
Genus	47	47
Genus +	53	53
Pf +, Pv −	14	14
Pf −, Pv +	35	33
Pf +, Pv +	2	2
Pf −, Pv −	2 (false −)	4 (false −)
Total	100*	100*

*Pf* *P. falciparum*, *Pv* *P. vivax*, + Positive, − Negtive

*100 samples of potential *Plasmodium* infections, identified from pooling test positives from a *Plasmodium* genus molecular screen of 4,580 asymptomatic samples, were subject to genus confirmation and species identification runs by mCLIP-PCR and singleplex qPCR

In summary (Table 2), for species identification, if we take the results of the concordant samples and the sequencing results of the discrepant samples as the gold-standard [16 *P. falciparum* (Pf) positives and 84 Pf negatives, 39 *P. vivax* (Pv) positives and 61 Pv negatives], our mCLIP-PCR assay can diagnose asymptomatic *Plasmodium* infection with 100% sensitivity and specificity at genus level. At species level, it detected *P. falciparum* infection with 100% (16/16) sensitivity and 100% (84/84) specificity. For *P. vivax* infection, it detected at 94.9% (37/39) sensitivity and 100% specificity (61/61). In addition, mixed infection of both species was clearly identified (2/2). The positive and negative predictive values, as well as kappa coefficient of mCLIP-PCR assays are provided in Table 2.

**Table 2:** The performance of multi-section CLIP-PCR versus qPCR for *Plasmodium* genus and species assay

	Multi-section CLIP-PCR performance, % (95% confidence interval)				Kappa coefficient
	Sensitivity	Specificity	Positive predictive value	Negative predictive value	
*Plasmodium*	100 (91.6–100)	100 (90.6–100)	100 (91.6–100)	100 (90.6–100)	100
*P. falciparum*	100 (75.9–100)	100 (94.6–100)	100 (75.9–100)	100 (94.6–100)	100
*P. vivax*	94.9 (81.–99.1)	100 (92.6–100)	100 (88.3–100)	96.8 (88.0–99.4)	95.8

## Discussion

This report describes a high-throughput, quantitative molecular approach to identify both genus and species *Plasmodium* RNA directly from blood or DBS without RNA extraction and reverse transcription. Designed for large-scale screening for elimination, the 2-tiered approach uses 96-well plate format in an ELISA-like workflow that can be accomplished in 3–4 h.

The multi-section sandwich approach described in this study enabled cooperative target capture that resulted in not only detection of both genus and species *Plasmodium* without extraction, but also dramatically accelerated hybridization, cutting down the capture time from overnight to 30 min with similar performance. After capture, all impurities or inhibitors from blood are washed away, without the issue of interfering with subsequent steps, which is a major concern for “direct PCR” approaches using modified polymerase [18, 24, 25]. The limits of detection of our mCLIP-PCR are 0.011 parasites/μl for genus *Plasmodium*, 0.102 parasites/μl for *P. falciparum* and 0.783 parasites/μl for *P. vivax*, which are similar to the sensitivity of RT-qPCR [35] and are among the best in published malaria molecular methods [7, 9, 12, 18, 21, 31, 35–37].

In a field screening mCLIP-PCR used only 575 tests to screen a total of 4,580 asymptomatic samples, and identified 53 positives that were confirmed by the standard singleplex TaqMan qPCR, including two mixed infections. In addition, two genus-positive samples with no species signal in TaqMan qPCR were identified as *P. vivax* by mCLIP-PCR (Table 1), demonstrating better sensitivity of the extraction-free mCLIP-PCR assay than the standard qPCR using extracted DNA in species identification. As a comparison, using “direct PCR” with modified polymerases on malaria field samples achieved only 93% sensitivity compared with using extracted DNA [24].

Mixed infections present a challenge to molecular amplification as template amount from different species may vary considerably [2]. Indeed, our results showed the possibility of false negatives if multiplex amplification is used, consistent with previous studies on mixed infections where only the more abundant parasite is amplified when the concentration ratio is high [10, 12]. One study compared a qPCR method of singleplex species identification [32] with one using co-amplification of all species with genus-specific primers [31] on the same set of 119 samples, and found that in 10 of 14 mixed infections co-amplification only identified the predominant species [38]. Interestingly, both that study [38] and our data (Fig. 4) suggested drop-out of minor species occurred when the species concentration ratio went beyond 5. A possible solution may be multiplex targeting different species with independent primer sets that do not compete for binding to targets. Indeed, such one-tube multiplex qPCR for five human-infecting *Plasmodium* species has been reported [21, 37]. However potential problems may still exist due to competition for other reagents such as dNTP and enzymes, and/or interaction of different primers and the probes, resulting in lower sensitivity [13, 38, 39]. A study comparing three different methods using gel electrophoresis detection demonstrated that for mixed infections, one-tube multiplex PCR with independent species-specific primer sets is less sensitive than the 2-tiered “semi-nested” PCR where the 2nd PCR is multiplexed, and the latter is less sensitive than the nested PCR where the 2nd PCR is individual singleplex [12]. Thus, to achieve highest sensitivity at lowest cost, we decided on a 2-tiered approach with singleplex amplification in the 2nd round PCR. Importantly, the first genus screening involves only multiplex capture of all species targets but no co-amplification, thus avoiding the problem of minor species drop-off. Finally, the detection is via SYBR green melt curve analysis instead of hydrolysis probes, significantly lowering the cost.

One limitation of this study was that only two species identification were designed in the second round, potentially missing any mixed infection involving Pv or Pf and the three less common species capable of human infection. In our assay, all five species can be expected to be captured by our genus probes. Given the expected low prevalence of the three less common species in most countries, it is not clear whether the benefit of identifying a small number of such rare infections could justify the additional labor, time and cost resulting from extra 3 species tests for every second-round sample. Whether this is practical depends on the local prevalence situations and if necessary, those three malaria species can be detected by our assay by including in the probe mix extra capture hybridization probes specific to those three species, with distinctive tails for additional singleplex amplification in the second round.

Although, as an example, a malaria screen assay was developed in this study, there was nothing in the general methodology that is malaria-specific. The same approach can be used to develop screening assays for any infectious disease agents, provided a target nucleic acid sequence can be identified that contains both a genus-specific portion and a species-specific portion.

## Conclusions

We have developed a highly sensitive, high-throughput RNA screening technology that can identify *Plasmodium* genus and species in 4 h without RNA purification and reverse transcription. The assay in general can be used for large-scale infectious disease screening as a highly-sensitive and high-throughput approach with low cost and labor.

## Supplementary Information

**Additional file 1. Table S1.** Probe Sequences.

**Additional file 2. Table S2.** Additional tests at low concentration of targets for the Probit analysis.

**Additional file 3. Table S3.** Results of genus and species identification of mixed- or single-infection blood samples using singleplex mCLIPPCR or singleplex Taqman qPCR.

## Data Availability

All data generated during this study are included in this published article.

## Abbreviations

CLIP- PCR: Capture and ligation probe-PCR
CP: Capture probes
Cq: Quantification cycle
CT: Cycle threshold
DBS: Dried blood spot
DP: Detection probes
ddPCR: Droplet digital PCR
ELISA: Enzyme linked immunosorbent assay
LOD: Limit of detection
mCLIP-PCR: Multi-section CLIP-PCR
qPCR: Quantitative PCR
RDT: Rapid diagnostic test
