# Supplementary material for: A direct, sensitive and high-throughput genus and species-specific molecular assay for large-scale malaria screening
Source: Infect Dis Poverty. 2022 Mar 7;11:25. doi: 10.1186/s40249-022-00948-2 (PMC8900325; doi:10.1186/s40249-022-00948-2)
Supplement: Supplementary file 1 — Additional file 1. Table S1. Probe Sequences. [file 40249_2022_948_MOESM1_ESM.docx]

Probe Sequences

| Species | Primer or probe | Sequence (5’–3’) |
| --- | --- | --- |
| Plasmodium spp. | Genus-CP1 | ACTTTTCGGCGGAGGAA |
|  | Genus-CP2 | TCACGATATATATTGATAAAGATTACCTAC |
|  | Genus-DP1 | TGTAAAACGACGGCCAGTTTTTTAGCACAATCTGATGAATCATGCT |
|  | Genus-DP2 | P-TACTAGGCATTCCTCGTTCAAGA |
|  | Genus-DP3 | P-TTAATAATTGCAATAATCTATCCCCATTTTTGGTCATAGCTGTTTCCTG |
|  | Genus-Primer-F | TGTAAAACGACGGCCAGT |
|  | Genus-Primer-R | CAGGAAACAGCTATGACC |
| P. falciparum | Pf-CP1 | GAATACTCGCCCCAGAACC |
|  | Pf-CP2 | CAAAGACTTTGATTTCTCATAAGG |
|  | Pf -DP1 | CGCAAATGGGCGGTAGGCTTTTTTGTTACTGAAGGAAGCAATCTAAAAGTC |
|  | Pf -DP2 | P-ACCTCGAAAGATGACTTTTATTTT |
|  | Pf -DP3 | P-TAACACTTTCATCCAACACCTAGTTTTTTCCTCGACTGTGCCTTCTA |
|  | Pf-Primer-F | CGCAAATGGGCGGTAGGC |
|  | Pf-Primer-R | TAGAAGGCACAGTCGAGG |
| P. vivax | Pv-CP1 | GGGAAAGGGAAACTGTTACG |
|  | Pv-CP2 | GCCGCTAATTAGCAGGTTAAG |
|  | Pv-DP1 | TGTATCTTATGGTACTGTAACGTTTTTTTATCCCAATATTTTCTTCGAAGTA |
|  | Pv-DP2 | P-AGCAAATCAACCGAATTCAGT |
|  | Pv-DP3 | P-CCCACGTAAGAATATATCGTATTTTTTTTTGGGACAGCCTATTTTGCTAG |
|  | Pv-Primer-F | TGTATCTTATGGTACTGTAACG |
|  | Pv-Primer-R | CTAGCAAAATAGGCTGTCCC |

Target-complementary sequences of the probes binding to the Plasmodium genus-specific section and P. falciparum- or P.vivax-specific section of the target. ”P” in DP2 and DP3 sequences indicates 5’ phosphate. The PCR primer sequences are also included, as well as the tail sequences in the detection probes (DPs).
