# Supplementary material for: A direct, sensitive and high-throughput genus and species-specific molecular assay for large-scale malaria screening
Source: Infect Dis Poverty. 2022 Mar 7;11:25. doi: 10.1186/s40249-022-00948-2 (PMC8900325; doi:10.1186/s40249-022-00948-2)
Supplement: Supplementary file 2 — Additional file 2. Table S2. Additional tests at low concentration of targets for the Probit analysis. [file 40249_2022_948_MOESM2_ESM.doc]

|  | Parasites/μL | Total Replicates | Positive Number |
| --- | --- | --- | --- |
| Genus | 0.032 | 54 | 54 |
|  | 0.01 | 54 | 51 |
|  | 0.0038 | 54 | 31 |
| P. vivax | 2.6 | 54 | 54 |
|  | 0.86 | 54 | 52 |
|  | 0.29 | 54 | 40 |
|  | 0.096 | 54 | 24 |
| P. falciparum | 0.29 | 54 | 54 |
|  | 0.096 | 54 | 50 |
|  | 0.032 | 54 | 24 |

Additional tests at low concentration of targets for the Probit analysis
