# Supplementary material for: A direct, sensitive and high-throughput genus and species-specific molecular assay for large-scale malaria screening
Source: Infect Dis Poverty. 2022 Mar 7;11:25. doi: 10.1186/s40249-022-00948-2 (PMC8900325; doi:10.1186/s40249-022-00948-2)
Supplement: Supplementary file 3 — Additional file 3. Table S3. Results of genus and species identification of mixed- or single-infection blood samples using singleplex mCLIPPCR or singleplex Taqman qPCR. [file 40249_2022_948_MOESM3_ESM.doc]

Results of genus and species identification in singleplex assays of mCLIP-PCR

| mock mixed infections | genus | Species-Pv | Species-Pf |
| --- | --- | --- | --- |
| Pf : Pv = 1 : 1 | Pos | Pos | Pos |
| Pf : Pv = 1 : 3 | Pos | Pos | Pos |
| Pf : Pv = 1 : 5 | Pos | Pos | Pos |
| Pf : Pv = 1 : 7 | Pos | Pos | Pos |
| Pf | Pos | Neg | Pos |
| Pv | Pos | Pos | Neg |
| Healthy control | Neg | Neg | Neg |

Results of genus and species identification in singleplex assays of TaqMan qPCR

| mock mixed infections | genus | Species-Pv | Species-Pf |
| --- | --- | --- | --- |
| Pf : Pv = 1 : 1 | Pos | Pos | Pos |
| Pf : Pv = 1 : 3 | Pos | Pos | Pos |
| Pf : Pv = 1 : 5 | Pos | Pos | Pos |
| Pf : Pv = 1 : 7 | Pos | Pos | Pos |
| Pf | Pos | Neg | Pos |
| Pv | Pos | Pos | Neg |
| Healthy control | Neg | Neg | Neg |
